# Notes on methods and analysis of ground nests of apoid wasps (Hymenoptera, Apoidea)

**DOI:** 10.3897/BDJ.13.e161769

**Published:** 2025-10-13

**Authors:** Piotr Olszewski, Petr Bogusch

**Affiliations:** 1 Natural History Museum, Faculty of Biology and Environmental Protection, University of Lodz, Lodz, Poland Natural History Museum, Faculty of Biology and Environmental Protection, University of Lodz Lodz Poland; 2 Univerzita Hradec Králové, Hradec Kralove, Czech Republic Univerzita Hradec Králové Hradec Kralove Czech Republic

**Keywords:** Hymeoptera, apoidea wasps, methodology, architectonic nests, ethology

## Abstract

A large portion of species of apoid wasps (Spheciformes) place their nests underground. While nesting biology and structure of nests of cavity-nesting species are often studied, searching for nests in the ground is usually interpreted as very difficult or impossible. We present a simple and practical method employing plant stems to explore the profile (direction of inclination) and to measure their excavation depth and extent. This method is compared with other previously known methods for studying nests placed in ground. The paper also includes comments on the nest search technique.

## Introduction

Ground-nesting apoid wasps (Spheciformes) represent a fascinating group of insects that play a key role in ecosystems, particularly in regulating the populations of other organisms and supporting pollination processes. Most of these solitary wasps build their nests in soil, which provides a protected environment, crucial for the development of their offspring (Bohart and Menke 1976). Nesting in underground spaces offers several ecological advantages, including protection from predators and environmental factors, as well as maintaining optimal temperature and humidity conditions for egg development (Olszewski et al. 2024). In addition to their role in pollination, ground-nesting digger wasps are important for maintaining biodiversity by controlling pest populations, especially other insects (Kazenas 2001). Understanding the nesting behaviour and ecology of these wasps is essential to assess their impact on both natural ecosystems and agricultural systems, where pollinators play a vital role in crop production (Kazenas 2001). Research on the nesting technique of apoid wasps is mainly conducted using excavation and casting techniques (Linsley et al. 1952, Jones et al. 1998, Marinho et al. 2018, Antoine and Forrest 2021, Tschanz et al. 2023) or using observation chambers in laboratory conditions (Michener et al. 1955, Leonard and Harmon-Threatt 2019, Antoine and Forrest 2021).

The main problem with excavation and casting techniques is their destructive impact and time-consuming nature (Antoine and Forrest 2021). In most substrates, nest entrance and burrow usually do not persist long and climatic conditions (wind, rain and other) usually destroy both and it is thus impossible to find the brood cells several days after the female finishes its nest. Moreover, many ground-nesting species of Hymenoptera currently lack nesting opportunities (see Bogusch et al. (2020)) and so small patches of bare soil are often settled by many females of different species in a short time. In this case, random digging cannot lead us to the brood cells of the nest we want to excavate. Using observation chambers needs much space and is quite expensive. Furthermore, studied species may behave differently in laboratory conditions compared to their natural habitats and the use of observation chambers can alter their behaviour and often prevent successful reproduction (Michener et al. 1955, Leonard and Harmon-Threatt 2019, Antoine and Forrest 2021). The paucity of methodological data for studying the nesting biology of ground-nesting wasps has been both a challenge and an incentive to share personal observations and insights gained during research on the ethology of this group of insects. This article aims to provide guidelines for conducting research on the ethology of apoid wasps and analysing their nesting biology. The method of nest analysis proposed in this work has been successfully applied in our previous study and in several other ongoing studies. Although the use of plant stems to explore burrow structure has been mentioned in earlier works (e.g. Linsley et al. (1952)), its application to locate and study apoid wasp nests remains relatively underutilised.

## Materials and Methods

**Figure 1.**
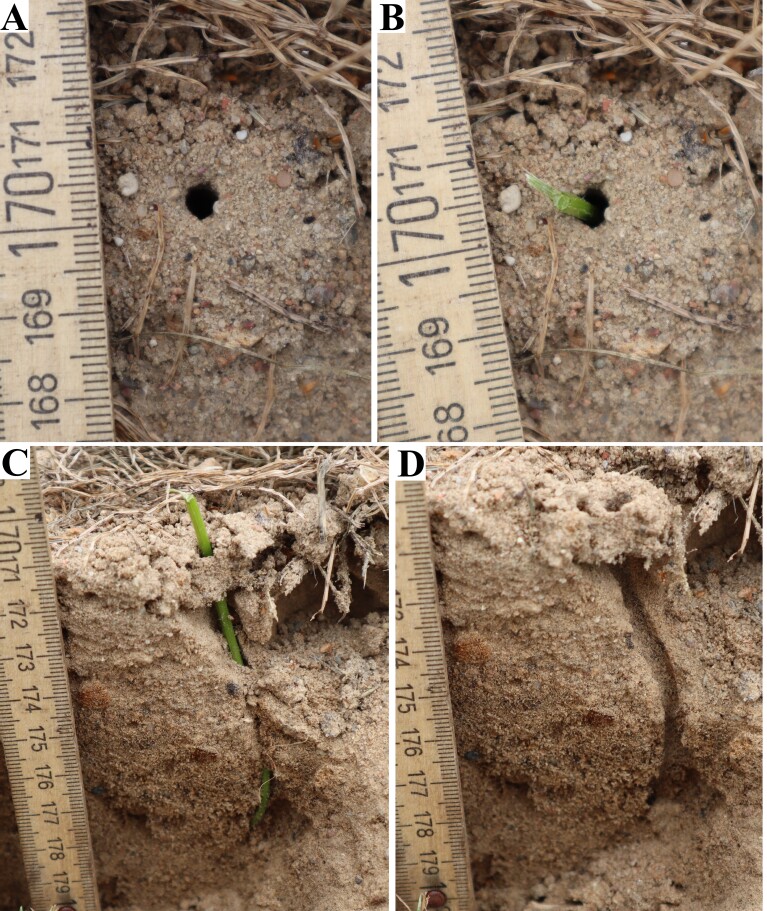
Analysis of the nest of *Cerceris quinquefasciata* (Rossi, 1792). **A** Top view of the nest entrance; **B** Top view of the nest entrances with a stem; **C** Lateral view of the nest with a stem; **D** Lateral view of the nest.

**Figure 2.**
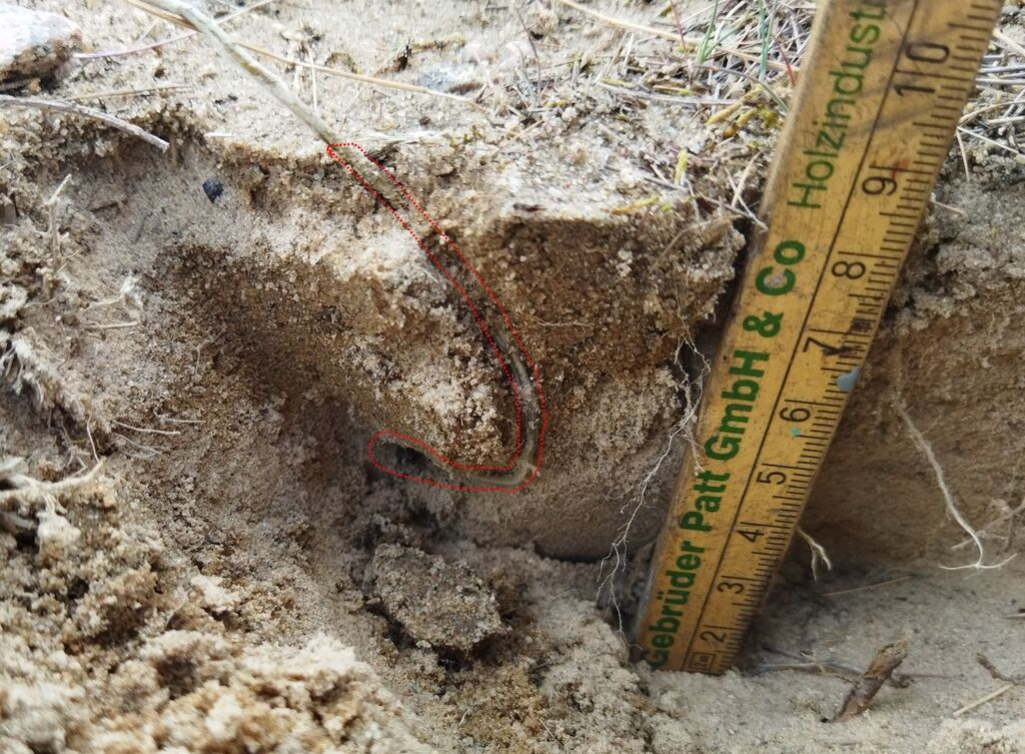
Analysis of the nest of *Oxybelus haemorrhoidalis* Olivier, 1812.

The ideas for analysing the structure of ground nests were inspired by multiple attempts and reflections in the field. The presented method is the result of long-time attempts. The testing of the method was done in 2020–2024 by excavating nests of the following species: *Alysson spinosus* (Panzer, 1801); *Cerceris quinquefasciata* (Rossi, 1792) (Fig. 1); *Dryudella stigma* (Panzer, 1809); *Lindenius albilabris* (Fabricius, 1793); *L. pygmaeus armatus* (Van der Linden, 1829), *Oxybelus bipunctatus* Olivier, 1812; *O. quatuordecimnotatus* Jurine, 1807; O. *haemorrhoidalis* Olivier, 1812 (Fig. 2); *O. trispinosus* (Fabricius, 1787); *O. uniglumis* (Linnaeus, 1758), *O. variegatus* Wesmael, 1852 and *Pterocheilus phaleratus* (Panzer, 1797). Depending on the diameter of nesting burrows, appropriately selected flexible stems, a water sprayer and a spatula are required; alternatively, a metal tablespoon can also be effective. Minimal cost, quick implementation, as well as widespread availability and ease of use ensure significant advantages for researchers of varying skill levels. During the fieldwork, once the nests have been located, it is helpful to set up a camera or use a mobile phone and notebook to record data.

## Results

### Searching for nests and observations

While searching in open areas on foot, we can often unknowingly destroy various apoid wasp nests. Some species cover their entrance with sand, while provisioning the cell (protection against kleptoparasites), which makes them completely invisible (e.g. *Oxybelus haemorrhoidalis* Olivier, 1812; *O. uniglumis*, *O. argentatus* Curtis, 1833; *O. bipunctatus*) (Andrietti et al. 2013). Insects remember the topography of the area around the nest entrance and use visual landmarks for navigation, so even slight movements of small elements can prevent the female from returning to the nest (Zeil 1993). Good results are achieved by observing in a sitting position (minimum 30 minutes) and gradually moving a few metres away and repeating the procedure.

### Nest analysis

The presented method of nest structure analysis seems to be relatively simple and accessible in the field. Depending on the respective prerequisites (burrow diameter), a suitable plant stem is used (the more flexible the better). It is important that the thickest part of the stem is less than half the diameter of the main burrow. Once the right plant has been located, remove its leaves right at the stem and measure its length. Then carefully insert the end of the stem into the burrow and gently push it until you feel resistance. Depending on the inclination angle and the length of the stem placed into the substrate, moisten the surface appropriately with a sprinkler and start digging. If the nest burrow is oblique, the direction of digging should be dictated by the angle that the stem forms with the substrate (Figs 1, 2). The inserted stem acts as a digging direction and, importantly, filling with sand does not cause any major losses. Use a spatula for digging. The digging itself should consist of carefully driving the blade of the spatula about 5 mm into the substrate, at a distance of no less than 1 cm from the entrance to the main burrow. The excavated material should be placed into a container so that a correct final interpretation can be made.

Additionally, a relatively large vertical hole was sometimes excavated at a short distance (about 10 cm) from the nest entrance, followed by careful scraping away of soil along the inserted stem to expose the burrow walls. Although this approach is more time-consuming and labour-intensive, it provides valuable detailed information on nest architecture and complements the stem insertion method.

## Discussion

Monitoring apoid wasp nests in the ground requires the use of a variety of methods to obtain a complete picture of their behaviour and social dynamics. The choice of the appropriate method depends on the purpose of the study, the available equipment and the type of stingers being studied. Visual observation, traps, cameras and labelling are basic techniques that, when combined, can provide very valuable data on the lives of these insects. The small number of methodological studies of ground nest structures (Marinho et al. 2018) has encouraged us to share information on this topic. Current methods of analysing ground nests involve casting techniques: plaster (Linsley et al. 1952, Michener et al. 1955, Norden et al. 1994), molten metal alloys, paraffin wax (Linsley et al. 1952), bioplastic (Howell 1960), fiberglass resin (Chapman et al. 1990) and liquid latex (Jones et al. 1998) and excavation techniques: cylindrical rubber refills (Marinho et al. 2018). The greatest limitation of the casting methods is that they are time-consuming, invasive and problematic for small nest structures in sandy substrates. Another field technique occasionally used in excavating bee nests is the so-called 'dusting' method, in which a brightly coloured or white powder (e.g. talc) is gently blown into the burrow using a small rubber bulb syringe. The powder helps to trace the burrow's trajectory and detect blockages. Although widely known amongst practitioners, this approach is not extensively documented in peer-reviewed literature (Smith et al. 2025). We would like to compare excavation techniques and compare our method with cylindrical rubber refills.

It is important to acknowledge that methods for studying underground nests have a long history. The seminal study of Malyshev (1931) provided a comprehensive overview of a wide range of approaches. In addition to describing the use of rubber rods for nest excavation, Malyshev gave detailed accounts of procedures for excavating nests, locating branch burrows and cells, tracing the sequence of preparing cells by bees and wasps and the step-by-step methods for opening and examining underground nests. He also outlined procedures for preserving nests after their removal from the soil. This pioneering study, therefore, laid the foundation for much of the methodological and biological research on nest structures in Apoidea. Later, Radchenko and Pesenko (1994) provided comprehensive overviews of casting techniques. Some species of bees, such as certain *Andrena* spp., are known to excavate deep or long nest burrows exceeding 30 cm (Pesenko et al. 1980, Westrich 2018), which makes non-invasive methods like stem probing insufficient for studying their nests. Therefore, our method is more suitable for apoid wasps and bee species with relatively shallow or short nesting tunnels.

However, it is worth noting at the outset that an important aspect in analysing the effectiveness of these methods is the type of substrate, the diameter of the nest burrow and the inclination angle of the burrow. Rubber, although flexible, is usually more rigid and cannot easily fit into small holes at large angles of inclination. When the angle of inclination of the burrow is large, the rubber may encounter resistance, which can cause it to block. On the contrary, if the plant stem used in our method is sufficiently flexible, it can easily pass through various burrow systems. The thin end of the stem and its elasticity at larger angles of inclination allow for easier insertion and sliding. Previously, we used stems of *Chenopodium* L. and *Veronica spicata* L. Compared to all other known methods, the advantage of the presented method is that it is cost-free and almost completely non-invasive and can be used for further breeding. The presented method allows for a fairly easy analysis of nests regardless of the type of substrate and nest burrow diameter. In some cases (when the nest burrow is non-linear in cross-section), the nest should be presented in three dimensions. Cryptic species complexes pose a serious challenge to taxonomists and change the understanding of species variability. Recent estimates show that, for each insect species described, based on morphological differences, there are, on average, about three cryptic species (Li and Wiens 2023). In view of this information, it is important to be aware of recording all details of the observations in all aspects related to nesting biology. Further, current ongoing projects, focused on conservation of bees, resulted all in the same conclusion that taxonomy and nesting and foraging ecology of this group are understudied. This fact is one of the main reasons of the situation that we cannot appropriately conserve most bee species because their ecology is unknown (Nieto et al. 2014, Reverté et al. 2023). Recent studies emphasise that understanding nesting behaviour is crucial for effective conservation planning and management of wild bee populations (Smith et al. 2025).

While recent studies have emphasised the importance of nesting behaviour for conservation, it is equally important to recognise the evolutionary and phylogenetic value of such research. Studies of nesting biology, alongside morphological and molecular data, have provided critical insights into the origins and diversification of bees. For example, analyses of nearly identical nesting features in Ctenoplectra (Ctenoplectrini) and Tetrapedia (Tetrapediini) demonstrated their close relationship (Radchenko 1992, Radchenko 1996), a conclusion later supported by molecular phylogenetic studies (Bossert et al. 2019). Moreover, based on the nesting biology of oil-collecting bees, it was proposed that corbiculate bees originated from this group (Radchenko 1996), a hypothesis also corroborated by molecular evidence (Martins et al. 2014). More broadly, the reconstruction of the “proto-bee” carried out by Radchenko and Pesenko (1996) fundamentally changed our understanding of the ancestral traits of bees and this concept has since been widely accepted in apidology (Michener 2000, Michener 2007). Thus, the study of nesting biology is not only crucial for applied aspects such as conservation, but also represents a cornerstone for reconstructing the evolutionary pathways of bees and their close relatives, the apoid wasps.

Our methodology is useful in most substrates, where apoid wasps place their nests. Moreover, excavating even shallow nests in stony or rocky substrate can also bring difficulties, as well as excavating nests in very loose substrate (sand or gravel). As most species of apoid wasps nesting underground do not have nests deeper than 20-30 cm (see Blösch (2000)), our method is thus good for studies of nesting biology of a large portion of species of this group.
